# Simvastatin improves mitochondrial respiration in peripheral blood cells

**DOI:** 10.1038/s41598-020-73896-2

**Published:** 2020-10-12

**Authors:** Jon Ambæk Durhuus, Svenja Hansson, Thomas Morville, Anja Birk Kuhlman, Tine Lovsø Dohlmann, Steen Larsen, Jørn Wulff Helge, Maria Angleys, Alba Muniesa-Vargas, Jens R. Bundgaard, Ian David Hickson, Flemming Dela, Claus Desler, Lene Juel Rasmussen

**Affiliations:** 1 Department of Cellular and Molecular Medicine, Center for Healthy Aging, University of Copenhagen, Copenhagen, Denmark; 2 The HNPCC Register, Clinical Research Center, Copenhagen University Hospital, Hvidovre, Denmark; 3 Department of Biomedical Sciences, XLAB, Center for Healthy Aging, University of Copenhagen, Copenhagen, Denmark; 4 Clinical Research Centre, Medical University of Bialystok, Bialystok, Poland; 5 Department of Clinical Biochemistry, University of Copenhagen, Copenhagen, Denmark; 6 Department of Cellular and Molecular Medicine, Center for Healthy Aging, Center for Chromosome Stability, University of Copenhagen, Copenhagen, Denmark; 7 Department of Geriatrics, Bispebjerg-Frederiksberg Hospital, Copenhagen, Denmark

**Keywords:** Molecular medicine, Mitochondria

## Abstract

Statins are prescribed to treat hypercholesterolemia and to reduce the risk of cardiovascular disease. However, statin users frequently report myalgia, which can discourage physical activity or cause patients to discontinue statin use, negating the potential benefit of the treatment. Although a proposed mechanism responsible for Statin-Associated Myopathy (SAM) suggests a correlation with impairment of mitochondrial function, the relationship is still poorly understood. Here, we provide evidence that long-term treatment of hypercholesterolemic patients with Simvastatin at a therapeutic dose significantly display increased mitochondrial respiration in peripheral blood mononuclear cells (PBMCs), and platelets compared to untreated controls. Furthermore, the amount of superoxide is higher in mitochondria in PBMCs, and platelets from Simvastatin-treated patients than in untreated controls, and the abundance of mitochondrial superoxide, but not mitochondrial respiration trends with patient-reported myalgia. Ubiquinone (also known as coenzyme Q10) has been suggested as a potential treatment for SAM; however, an 8-week course of oral ubiquinone had no impact on mitochondrial functions or the abundance of superoxide in mitochondria from PBMCs, and platelets. These results demonstrate that long-term treatment with Simvastatin increases respiration and the production of superoxide in mitochondria of PBMCs and platelets.

## Introduction

Hypercholesterolemia is a major risk factor of atherosclerosis leading to cardiovascular disease (CVD) including heart attack, and stroke^1^. Statins are a class of drugs that inhibit 3-hydroxy-3-methylglutaryl coenzyme A (HMG-CoA) reductase, which effectively reduce hypercholesterolemia^1^, and associated CVD and CVD-related mortality^2–4^. Statins are some of the most commonly prescribed drugs worldwide and more than 10% of the population is treated with statins in both Scandinavia and the US^5–7^. Statins are generally well-tolerated, despite some notable adverse effects including the very rare, but fatal rhabdomyolysis^8^. A more frequent side effect is myalgia, which is reported by 10–15% of statin users^9,10^. Statin-Associated Myopathy (SAM) takes the form of mild to moderate symptoms of skeletal muscle discomfort, pain, and cramps, as reported in a number of observational studies^9,10^. There are several mechanisms in the skeletal muscle proposed and demonstrated to be involved in SAM which have been reviewed in detail elsewhere^11–13^. SAM can discourage physical activity, which in itself may result inadvertently result in an increase in cholesterol levels^14^. Furthermore, adverse side effects and concern over statin-induced side effects can lead to discontinuation of the treatment, which is associated with an increased risk of myocardial infarct, and CVD-related death^10,15^. Therefore, it is imperative to understand the mechanism underlying SAM, so it can be mitigated or appropriately managed.

It has been suggested that statin use impairs mitochondrial respiration in muscle, blood, and liver^16–23^, which in turn has been suggested as the underlying cause of the myalgia^23^. Statins intended biological target is cholesterol by inhibition of HMG-CoA reductase in the mevalonate pathway. This inhibition also leads to reduced biosynthesis of mevalonate, farnesyl pyrophosphate, and ubiquinone (UQ). UQ, also known as coenzyme Q10, mediates the transport of electrons from complex I, and II to complex III in the electron transport chain (ETC)^24^. One hypothesis suggests that lower levels of UQ found in plasma, peripheral blood mononuclear cells (PBMCs), and skeletal muscle of statin users result in an impairment of the ETC, thereby reducing the rate of mitochondrial respiration^17,25–27^.

Here, we provide evidence that is not consistent with this hypothesis. On the contrary, we demonstrate that long-term Simvastatin therapy has the opposite effect, significantly improving mitochondrial respiration in patient-derived platelets, and PBMCs. We argue that the hypothesis of a negative relationship between statin usage, and mitochondrial function is based on suboptimal experimental setups, and we demonstrate that short-term Simvastatin therapy at a dose vastly exceeding the pharmacologically relevant dose does in fact impair mitochondrial respiration in human hepatocarcinoma cells cultured in vitro. Therefore, the effect of statins on mitochondrial respiration appears to be context-, and dose-dependent.

In this study, mitochondrial parameters were evaluated in patient-derived platelets, and PBMCs, with the aim to investigate statin-induced mitochondrial alterations. We used blood cells in this study because it is less invasive to access compared to muscle cells obtained from muscle biopsies. Moreover PMBCs and platelets are well-characterized surrogate models for assessing pharmacological effects on skeletal muscle mitochondrial function^28–33^. In addition to increasing mitochondrial respiration, we observed that Simvastatin increases production of mitochondrial superoxide, a representative reactive oxygen species (ROS) by-product of mitochondrial respiration, and that patient-reported myalgia increases with increasing abundance of superoxide, but does not trend with oxygen consumption rate (OCR) in mitochondria from patient-derived peripheral blood cells.

## Results

### Short term in vitro exposure to high concentration Simvastatin decreases mitochondrial respiration in Huh-7 cells

**Figure 1:**
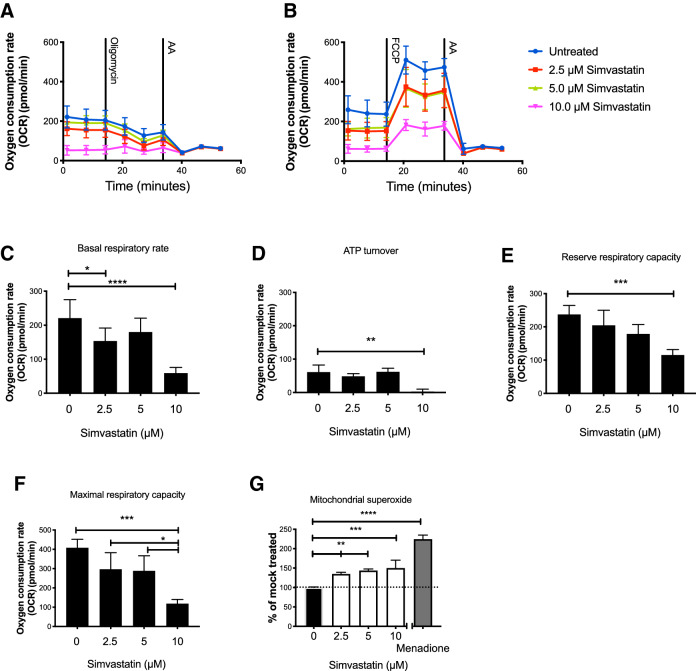
Oxygen consumption was quantified in Huh-7 cells in the presence of 0, 2.5, 5 or 10 µM Simvastatin, as indicated. OCR was measured before and after addition of the ATP synthase inhibitor oligomycin and complex III inhibitor, antimycin A (**A**), or before and after addition of the uncoupler FCCP and antimycin A (**B**); the data were used to calculate basal respiratory rate (**C**), ATP turnover (**D**), reserve respiratory capacity (**E**) and maximal respiratory capacity (**F**). Mitochondrial superoxide was quantified in Huh-7 cells treated with 0, 2.5, 5 or 10 µM Simvastatin; values were normalized to control (0 µM Simvastatin) and menadione was used as a positive control (**G**). Panels **A**–**F** show mean OCR ± standard deviation. Panel **G** shows the mean superoxide level ± standard deviation. At least three biological replicates were performed.

To investigate the effects of high dose Simvastatin treatment, the human hepatocyte-derived Huh-7 cell line was selected, as the effects of Simvastatin treatment are well described for this cell line^34,35^. In the present study, Huh-7 cells were exposed to 2.5–10 µM Simvastatin for 72 h in vitro, as this dose is widely used in the literature describing pleiotropic effects of Simvastatin treatment^36^. After 72 h of Simvastatin treatment, the effect on mitochondrial respiration was determined by evaluating basal respiratory rate, ATP turnover, reserve respiratory capacity, and maximal respiratory capacity (Fig. 1A,B,F). Basal respiratory rate is a measure of the rate of oxygen consumed by the cells when kept in media lacking inhibitors added (Fig. 1C). ATP turnover is measured as the decrease in rate of oxygen consumption after inhibition of the ATP synthase (Fig. 1D). This decrease is relative to ATP produced by oxidative phosphorylation. The reserve respiratory capacity is a measure of a theoretical extra capacity to produce ATP as a response to an increased energetic demand (Fig. 1E), and this has previously been correlated with the ability of cells to withstand periods of stress^37^. Reserve respiratory capacity is measured as the difference in OCR at basal, and that at maximal activity. Maximal respiratory capacity is a measure of total oxygen consumption possible by oxidative phosphorylation and it is measured as the difference in OCR following uncoupling by FCCP and after ETC inhibition by antimycin A (Fig. 1F).

Our results show that basal OCR in Huh-7 cells decreased significantly with increasing concentrations of Simvastatin, being 30% (*P* = 0.0126) or 75% (*P* < 0.0001) lower than control cells in the presence of 2.5 µM or 10 µM Simvastatin, respectively. ATP turnover (*P* = 0.0011), reserve respiratory capacity (*P* = 0.0005), and maximal respiratory capacity (*P* = 0.0002) were significantly decreased compared to controls in the presence of 10 µM Simvastatin but showed no effect at lower concentrations of Simvastatin. These results demonstrate that short term in vitro exposure to high-dose Simvastatin significantly impairs mitochondrial respiration in Huh-7 cells.

### Short term in vitro exposure to high dose Simvastatin increases production of mitochondrial superoxide production in Huh-7 cells

Mitochondrial superoxide was 1.35–1.50 fold higher in Huh-7 cells exposed to 2.5–10 µM Simvastatin than in untreated controls (Fig. 1G) (*P* = 0.01; *P* < 0.0021, and *P* = 0.0007 for 2.5, 5, and 10 µM Simvastatin, respectively). In cells exposed to 10 µM Simvastatin, superoxide production increased to 30% of the level induced by exposure to menadione, a level that could cause significant oxidative damage to nucleic acids, and other cellular macromolecules. These results indicate that in vitro exposure to high dose Simvastatin has the potential to cause significant respiratory and oxidative alterations.

### Establishment of the cohort

The effect of Simvastatin on mitochondrial respiration in human subjects was investigated in a cohort of 40 patients with at least 6 months history of Simvastatin use (20–40 mg per day), and 12 matched age-, weight-, BMI-, and body fat matched control subjects (Table 1). The concentration of white blood cells (WBC), neutrophils and monocytes were slightly, but significantly increased in Simvastatin users compared to controls (*P* = 0.034; *P* = 0.047, and *P* = 0.027 respectively) (Table 1). There was no difference in VO_2_ max levels between Simvastatin users and controls, indicating similar levels of fitness (data not shown).

**Table 1:** Characteristics.

	Control (n = 12)	Simvastatin (n = 40)
Male/female	6/6	(20/20)
Age (years)	61 ± 2	62 ± 1
Weight (kg)	83 ± 4	85 ± 3
BMI (kg/m^2^)	27 ± 1	28 ± 2
Body fat (%)	36 ± 3	36 ± 1
Cholesterol (mM)	6.0 ± 1.1	4.1 ± 0.5
WBC (10^9^ cells/L)	5.0 ± 0.3	5.8 ± 0.2 (*)
Platelets (10^9^ cells/L)	232 ± 12	238 ± 8
Neutrophils (10^9^ cells/L)	2.7 ± 0.2	3.3 ± 0.1 (*)
Lymphocytes (10^9^ cells/L)	1.7 ± 0.5	1.8 ± 0.4
Monocytes (10^9^ cells/L)	0.41 ± 0.03	0.50 ± 0.02 (*)

Values are mean ± SEM.

*BMI* body mass index, *WBC* white blood cells.

(*) Indicates a significant difference *P* < 0.05.

### Simvastatin usage is correlated with increased mitochondrial respiration

**Figure 2:**
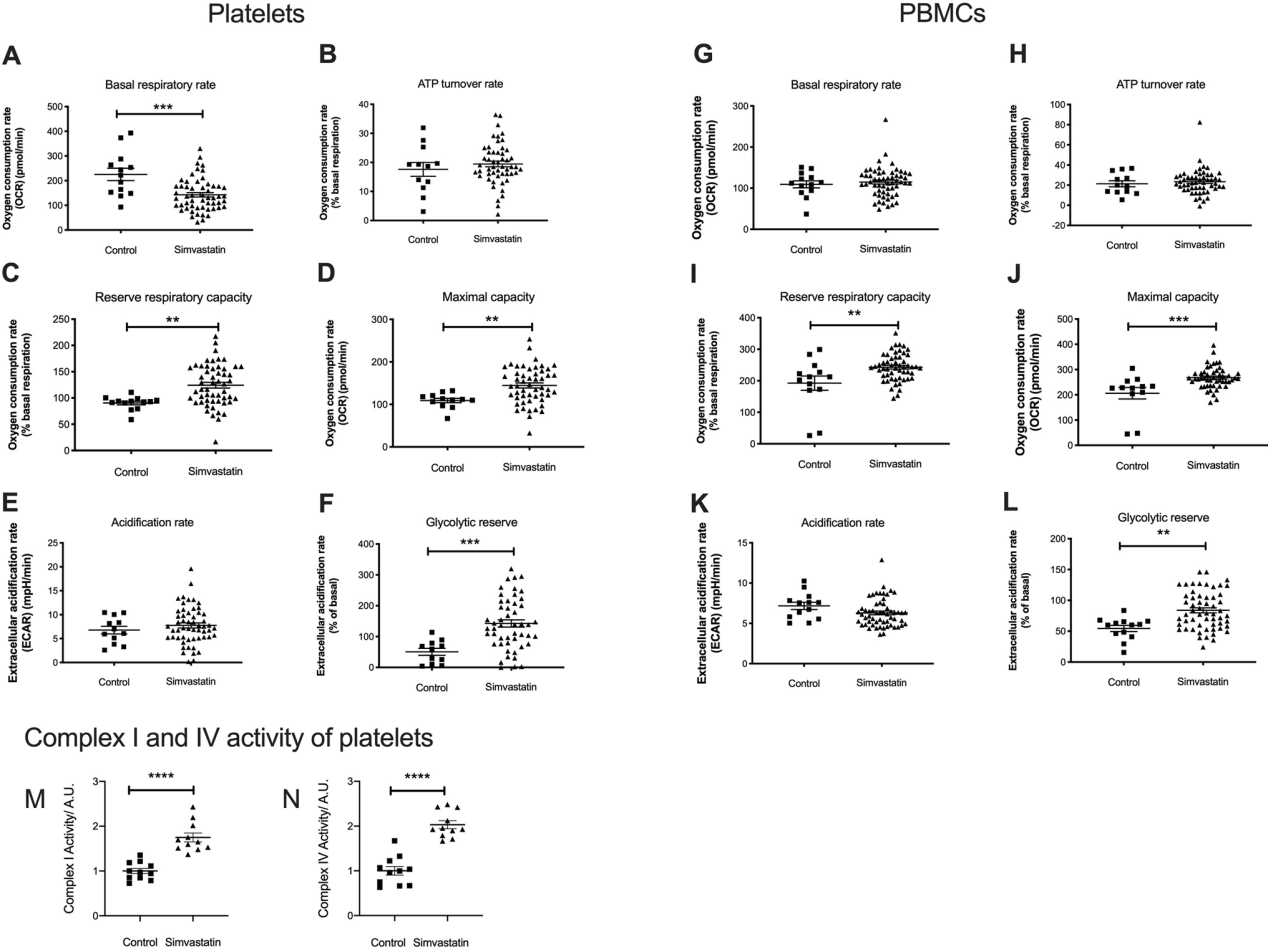
Platelets and PBMCs were isolated from Simvastatin users and controls as described in the text, and scatter plots are shown for the following mitochondrial respiratory parameters: basal OCR (**A** and **G**), ATP turnover (**B** and **H**), reserve respiratory capacity (**C** and **I**), maximal respiratory capacity (**D** and **J**), ECAR (**E** and **K**), and glycolytic reserve (**F** and **L**). Values for OCR were normalized to the basal OCR, and ECAR was normalized to control acidification rate. All values are shown ± standard error of the mean. Individual data points correspond to 13 controls and 58 Simvastatin users. An antibody-based dipstick assay was used to quantify active forms of complex I and complex IV subunits in platelets from Simvastatin users and controls (**M** and **N**). Individual data points correspond to eleven biological replicates.

Mitochondrial parameters were measured in PBMCs, and platelets from the cohort of Simvastatin users and controls. The results show a 63% decrease in basal OCR in platelets, but not in PBMCs, from Simvastatin users (*P* = 0.0003) (Fig. 2A,G). Reserve respiratory capacity was 1.37 fold higher in platelets, and 1.43 fold higher in PBMCs, respectively (*P* = 0.0038 and *P* = 0.0027) (Fig. 2C,I) in Simvastatin users than in controls. This indicates an improved ability to accommodate energy demand. No difference in ATP turnover rate was detected in platelets or PMBCs (Fig. 2B,H), demonstrating equivalent levels of oxygen used for ATP production. A significant increase in maximal OCR was observed in platelets (1.33 fold*, P* = 0.0071), and PMBCs (1.30 fold, *P* = 0.0004) (Fig. 2D,J) demonstrating an overall increase of mitochondrial respiration. extracellular acidification rate (ECAR) values were similar in platelets, and PBMCs from Simvastatin users, and controls (Fig. 2E,K), while glycolytic reserve was 2.82 fold, and 1.53 fold higher in platelets and PBMCs, respectively (*P* = 0.0007 and *P* = 0.0015) (Fig. 2F,L). Glycolytic reserve is the difference in ECAR in the presence and absence of an ATP synthase inhibitor. The glycolytic reserve is, therefore, a measure of the glycolytic capability of a cell to respond to an inhibition of oxidative phosphorylation. Assuming that the glycolytic capacity is unaltered, a higher glycolytic reserve indicates a higher dependence on oxidative phosphorylation in platelets and PBMCs from Simvastatin users than controls. Simvastatin users were stratified into users with (N = 14), and without (N = 17) self-reported myalgia. We found no significant association between myalgia, and any measured parameter of mitochondrial respiration or glycolysis (Figure S1).

### Complex I and complex IV activity is higher in Simvastatin users

To describe the functional background for the measured mitochondrial attributes, a dipstick assay was used to quantify the activity of ETC complexes I and IV in platelets. This assay detected 1.70 fold, and 1.94 fold more active ETC complex I and complex IV, respectively, in Simvastatin users than in controls (*P* < 0.0001) (Fig. 2M,N). These data are consistent with above results, showing higher OCR, and reserve respiratory capacity in Simvastatin users than in controls (Fig. 2I,J).

### Mitochondrial DNA content is similar in PBMCs from Simvastatin users and controls

The amounts of mitochondrial and nuclear DNA (mtDNA, nDNA) were quantified by real-time quantitative PCR (qPCR) in PBMCs from 20 Simvastatin users, and eight controls (Figure S2) and the mtDNA:nDNA ratio was calculated. There was no significant difference in this ratio in PBMCs from Simvastatin users and controls. The mtDNA, and nDNA content was not quantified in platelets, because platelets are anuclear.

### Mitochondrial superoxide is higher in Simvastatin users than in controls

**Figure 3:**
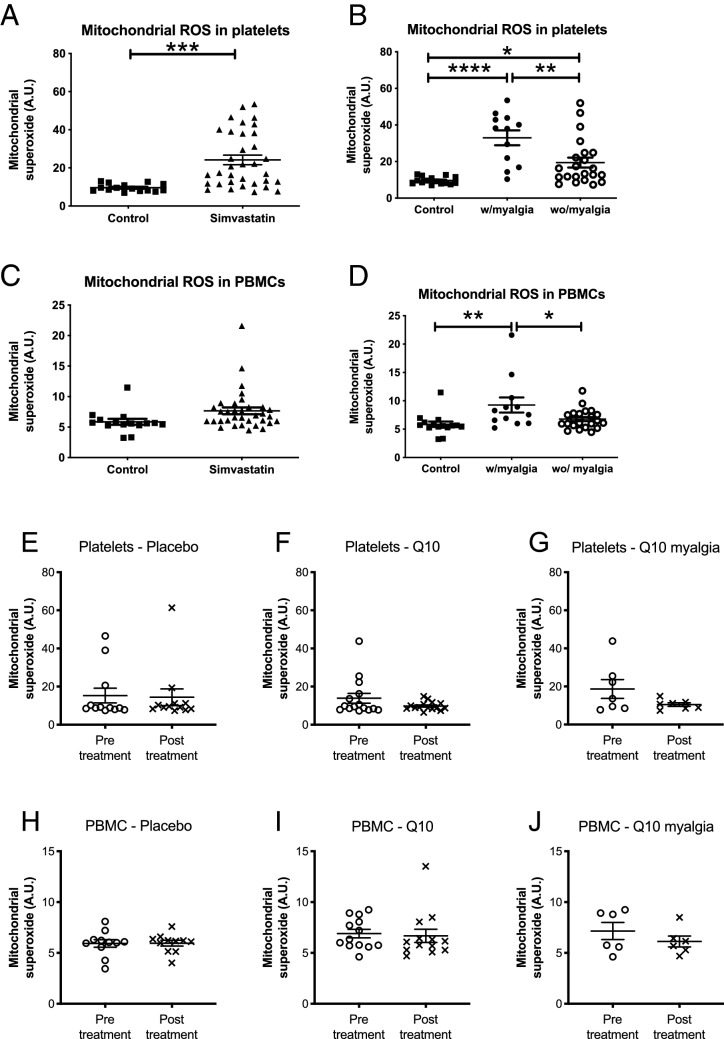
Mitochondrial superoxide was quantified in platelets and PBMCs from Simvastatin users (N = 34) and controls (N = 15) (**A** and **C**). Where indicated, Simvastatin users were stratified according to presence (N = 12) or absence (N = 22) of self-reported myalgia (**B**, **D**). Values shown are mean ± S.E.M. A subset of 24 Simvastatin users were dosed with placebo (**E**, **H**; N = 11) or Q10 (**F**, **G**, **I**, J; N = 13) for 8 weeks. Platelets (**E**–**G**) and PBMCs (**H**–**J**) were isolated and analyzed for mitochondrial superoxide before and after treatment, as indicated. Q10-treated patients were stratified according to the presence (N = 6) or absence of myalgia, as indicated (**G**, **J**). Mitochondrial superoxide was measured in arbitrary units (A.U.) and values shown are mean ± S.E.M.

Mitochondrial superoxide was measured as a representative of mitochondrial produced ROS in platelets and PBMCs from Simvastatin users and controls. The results indicated a 2.52-fold (*P* = 0.0003) increase in superoxide in platelets, but no significant difference in superoxide was detected in PBMCs from Simvastatin users, and controls (Fig. 3A,C). Superoxide level was compared in Simvastatin users with myalgia (N = 12), and Simvastatin users without myalgia (N = 22). The results revealed that the elevated superoxide levels are strongly associated with reported myalgia, with a 3.43- and 1.58-fold higher level of superoxide in platelets and PBMCs, respectively in Simvastatin users with SAM than in Simvastatin users without (Fig. 3B,D) (*P* < 0.0001; and *P* = 0.0075). Similarly, there was a significant decrease in superoxide in platelets and PBMCs when comparing Simvastatin users not reporting SAM to Simvastatin users reporting myalgia (*P* < 0.0035; and *P* = 0.045) (Fig. 3B,D).

### Ubiquinone has no effect on mitochondrial respiration or mitochondrial superoxide

To investigate the effects of UQ on mitochondrial respiration, 13 Simvastatin users were treated with 2 × 200 mg UQ per day for eight weeks, while 9 Simvastatin users were given a placebo^38^. Mitochondrial OCR, basal respiratory rate, ATP turnover, and reserve respiratory capacity were quantified before and after treatment with either UQ or placebo; however, the results revealed no significant differences in these mitochondrial parameters (Figure S3).

Similarly, the abundance of mitochondrial superoxide in platelets, and PBMCs was not significantly different before, and after dosing with UQ or placebo (Fig. 3E,F,H,I), irrespective of the presence or absence of SAM. These data strongly suggest that UQ, under the conditions tested here, has no impact on the level of mitochondrial superoxide in platelets or PMBCs (Fig. 3G,J).

## Discussion

Statins reduce hypercholesterolemia and are important for the prevention of CVD. However, statin use is frequently associated with myalgia leading to non-compliance with the recommended treatment protocol. To better understand the mechanisms underlying SAM, we assessed mitochondrial parameters and the abundance of superoxide in Simvastatin users, and matched controls, using platelets and PBMCs as a surrogate model for skeletal muscle^28–33^.

In contrast to previous studies^16–23^, we report here that statin usage is correlated with improved oxidative phosphorylation properties of a tissue. We demonstrate that reserve respiratory capacity, maximal capacity, glycolytic reserve, and complex I and IV activity are higher in platelets and PBMCs from Simvastatin users than in control subjects. At the same time, basal OCR was similar or lower in Simvastatin users than in controls, and cells of Simvastatin users demonstrated a higher glycolytic reserve. These results are consistent with our proposal that platelets and PBMCs from Simvastatin users demonstrate higher respiratory efficiency than control cells, in that they produce more ATP molecules for each oxygen molecule consumed. An increase of reserve respiratory capacity of PBMCs has been associated with gait speed, physical performance, muscle strength and muscle quality in adults and older adults^28,39^. The biological role of the increased reserve respiratory capacity as well as other increased aspects of mitochondrial respiration is unclear.

A previous study of hyperlipidemic patients treated for four weeks with 40 mg Simvastatin per day, demonstrated an approximately 50% increase of celullar oxidative consumption and a 25% increase of mitochondrial membrane potential of their polymorphonuclear leukocytes^40^. This supports our findings of a positive correlation between Simvastatin usage and ability of blood cells to perform mitochondrial respiration. However, it is not clear why we see an increased capacity for respiration. The complexes of the ETC can assemble into structures called supercomplexes, which modulates electron transport and ROS production (reviewed in^41^). Supercomplex formation is believed to be a very dynamic process and of great importance for regulation of mitochondrial respiration^42,43^. Recently, it has been demonstrated that endothelial cells changed their supercomplex formation in response to atorvastatin^44^. Supercomplex formation can be mediated by increased levels of ROS^45^. Conversely, it has been demonstrated that a change of supercomplex conformation resulting in a dissociation of complex I from the supercomplex results in an increased mitochondrial ROS production^46^. In this study we find an increased production of mitochondrial superoxide in platelets and PBMCs of Simvastatin users, and we can demonstrate an increased activity of complex I in these cells. Both factors support our suggestion of a regulatory involvement of supercomplexes, however, an elucidation to this requires a dedicated study.

As a caveat to this study, we acknowledge evidence for differential effects of statins on different tissues as has been seen demonstrated by others^47^; it is therefore possible that statin use improves various functions of mitochondria in blood cells, as reported here, while decreasing these in skeletal muscle. We and others, have reported a decrease of inflammation markers in response to long term statin treatment^38,48^. It is therefore possible that the altered mitochondrial characteristica of PBMCs is linked to lower levels of inflammation. This decrease is not reflected in levels of WBC, neutrophils and monocytes which are slightly elevated in Simvastatin users, but more work is needed to describe the role of statin modulated mitochondria in inflammation. Nevertheless, even though other reports suggest that mitochondrial respiration is impaired in muscle, blood, and liver of Simvastatin users^18–22^, we argue that these discrepancies could reflect different experimental approaches, and conditions used in previous studies. For example, the majority of published in vivo studies on mitochondrial function in statin users involved shorter periods of treatment (24 h–4 weeks), or in vitro exposures at doses up to 1000-fold higher than the physiologically-relevant dose, e.g. 2.5 to 10 µM vs*.* 1.6–4.3 nM Simvastatin^36,49^. Using Huh-7 cells exposed to 2.5–10 µM Simvastatin for 72 h, we also observed impaired respiration and increased production of mitochondrial superoxide, as reported previously by others. We therefore argue that there is an unmet need to investigate the mitochondrial respiration of both muscle and blood cells in persons receiving Simvastatin. Preferably in a cohort that is monitored since before the first Simvastatin administration and at least 6 month into the treatment.

Importantly, we report here that increased myalgia trend with a significant increase in mitochondrial superoxide in PBMCs and platelets from statin users, while myalgia did not trend with other measures of mitochondrial respiration. This finding agrees with a recent study demonstrating a 41% increase in mitochondrial superoxide in mouse skeletal myotubes exposed to atorvastatin^50^. The study also demonstrated a statin-induced increase in glutamate efflux mediated by the xC^-^ cysteine/glutamate antiporter, and argues that these factors could explain SAM^50^.

In a previous study on the same cohort, we demonstrated a five-fold increase in serum levels of UQ^51^. However, here we report that mitochondrial respiration and the abundance of superoxide in blood cells were similar in Simvastatin users dosed with UQ or placebo for 8 weeks independent of the presence or absence of SAM. Admittedly, the sample size for this experiment is very small (N = 7 Simvastatin users with myalgia); therefore, it remains possible that a beneficial effect of UQ on myalgia could be observed in a future study on a larger patient cohort. Nevertheless, other studies generally agree with our findings, indicating limited benefit of UQ towards SAM^38,52^.

In conclusion, this study demonstrates that long-term treatment with Simvastatin increases mitochondrial respiratory capacity in PBMCs and platelets. Reported side effects of Simvastatin usage are correlated to increased levels of mitochondrial superoxide and do not correlate with any mitochondrial respiratory parameters measured to date. Therefore, elevated mitochondrial superoxide in peripheral blood cells has potential as a biomarker for SAM.

## Methods

### Cell lines

The hepatocarcinoma cell line Huh-7 was cultured in a humidified 37 °C, 5% CO_2_ incubator, and maintained in Dulbecco’s Modified Eagle Medium (DMEM) with 10% foetal bovine serum (FBS), 1% penicillin, and streptomycin. Upon treatment with Simvastatin, cells were seeded in 6-well plates at 2 × 10^5^ cells/well 24 h before addition of Simvastatin. Cells were washed and incubated for 72 h in growth media containing 0, 2.5, 5 or 10 μM Simvastatin (Sigma-Aldrich, cat. No S6196).

### Participants

This study is part of the interdisciplinary project “Living with statins” (LIFESTAT)^7^. The participants used in this study were also examined in other studies^53–55^ including a study by Dohlman et al., where muscle tissue was studied in detail^16^. Informed consent have been obtained from all participants in this study. The experiments presented in this study was conducted based on two sub-studies from LIFESTAT; A cross-sectional study, and an interventional study. In the cross-sectional study statin-users with or without myalgia were compared to hypercholesteraemic controls (no therapy). The intervention consisted of eight weeks of UQ-therapy in Simvastatin-users, and controls, as described previously^38^. For practical reasons, the various cellular tests were performed on different population sizes. All procedures were approved by the Scientific Ethics Committee for the Capital Region of Denmark (H-2-2013-164), registered in Clinical Trials (NCT02250677), and conducted in accordance with the Helsinki Declaration.

### Platelet and PBMC purification

Venous blood was sampled in 6.0 mL BD Vacutainer blood collection tubes containing EDTA (BD Biosciences). The participants were fasted overnight and sampled the following morning. PBMCs were isolated as previously described^56^ with minor changes, including a platelet purification step as described^57^. Platelets, and PBMCs were used immediately for extracellular flux assay, and mitochondrial superoxide measurement, or cryopreserved in freezing medium (70% FBS, 25% Roswell Park Memorial Institute (RPMI) 1640, (Thermo Fisher Scientific- Life tech.), 5% DMSO) for later testing of ETC complex I, and complex IV activity as well as mtDNA levels by qPCR.

### SYSMEX haematology analyser

Blood samples were analysed for complete blood cell count, red blood cells, leukocytes, and platelets using a Sysmex XN automated haematology analyser XN (Sysmex Corporation, Kobe, Japan). Furthermore, platelets, and PMBCs were purified, and monocytes, lymphocytes, and neutrophils were counted in whole blood, and the PBMC fraction.

### Determination of mitochondrial respiration and extracellular acidification rate

OCR and ECAR were quantified using Agilent Seahorse Extracellular Flux (XF) technology on an XF Analyzer (Agilent Technologies). Cells were seeded in a Seahorse XF plate using Cell-Tak adherent (Corning) with 4 × 10^4^ Huh-7 cells, 2 × 10^7^ platelets or 6 × 10^5^ PBMCs per well, and resuspended in Seahorse assay media (Seahorse Bioscience, Agilent) containing 25 mM glucose, 2 mM pyruvate, 2 mM glutamine, adjusted to pH 7.4. OCR and ECAR was measured in the presence of oligomycin (1 µM for Huh-7 cells, and 0.5 µM for platelets and PBMCs) or Carbonyl Cyanide-4-(triFluoromethoxy) Phenylhydrazone (FCCP) (0.75 µM for Huh-7 cells, 0.9 µM for platelets, and 0.6 µM for PBMCs). All samples were then treated with 2 µM antimycin A as a control. Samples were measured as the median of 4 or 5 technical replicates.

### Determination of the specific activity of electron transport chain complex I and complex IV

Complex I (rotenone insensitive NADH-dehydrogenase specific), and complex IV dipstick assays (Abcam kits 109720, and 109876) were performed using cryopreserved platelets, as described previously^58^.

### Determination of mitochondrial produced superoxide

Mitochondrial superoxide production was quantified by flow cytometry (FACScalibur, BD Bioscience) using MitoSOX Red (Molecular Probes, Invitrogen). PBMCs and platelets were resuspended in 5 µM MitoSOX Red. After 10 min of incubation, cells were washed three times with PBS. MitoSOX Red was excited at 488 nm and data collected at FSC, SSC and 585/42 nm. The geometric mean fluorescence intensity values of the samples were obtained by subtracting the fluorescence of 50.000 control cells (not stained with MitoSOX) from the fluorescence of 50.000 MitoSOX stained cells. Menadione was used as a positive control (Sigma-Aldrich, cat. No M5750).

### Determination of relative mitochondrial DNA levels by quantitative PCR

Genomic DNA was extracted from 1 × 10^6^ cryopreserved PBMCs from Simvastatin-treated subjects with or without myalgia, and from controls. Extraction was performed using the GeneJET genomic DNA purification kit (Thermo Fish. Sci. Life). mtDNA to nDNA ratio was analyzed by qPCR using the StepOnePlus real-time PCR system (Applied Biosystems, and threshold (double delta Ct) values, as described previously^59^). tRNALeu (UUR) was the reference mitochondrial gene, and β-2-microglobulin (β2M) was the nuclear reference gene, as described previously^60,61^. The mtDNA/nDNA ratio was determined by threshold (double delta Ct) values as described^59^. All assays were performed in technical triplicate on a MicroAmp Fast Optical 96-Well Reaction Plate with MicroAmp Optical Adhesive Film (Thermo Sci.).

### Statistics

For comparison of mitochondrial respiration, ECAR, complex I, and IV activity, mtDNA content, and mitochondrial superoxide, significant difference between controls, and Simvastatin users was calculated using two-tailed unpaired Student’s t-test. For comparison of effect of increasing Simvastatin treatment on mitochondrial respiration, and superoxide levels, as well as between controls, Simvastatin users experiencing myalgia, and Simvastatin users not experiencing myalgia, single classification analysis of variance (ANOVA) was used. Assumptions of normality were checked by visual inspection prior to ANOVA. When the ANOVA indicated significant differences, Dunnett’s (mitochondrial respiration) or Tukey’s (mitochondrial superoxide) honestly significant method was used to test for differences. *P* values below 0.05 were considered significant. Results from Huh-7 cells are presented as mean ± SD, while results from human subjects are presented as mean ± SEM. Statistical analyses were performed using GraphPad Prism version 8.3 (GraphPad Software, La Jolla California USA).

## Supplementary information

Supplementary file1

Supplementary file2

## Data Availability

The authors declare that the data supporting the findings of this study are available within the article and its Supplementary Information files. The raw data for the figures and supplementary figures is presented in the Source Data file.
