# Supplementary material for: Simvastatin improves mitochondrial respiration in peripheral blood cells
Source: Sci Rep. 2020 Oct 12;10:17012. doi: 10.1038/s41598-020-73896-2 (PMC7550337; doi:10.1038/s41598-020-73896-2)
Supplement: Supplementary file 1 — Supplementary file1 [file 41598_2020_73896_MOESM1_ESM.docx]

**Simvastatin increases the efficiency of mitochondrial respiration in peripheral blood cells**

Jon Ambæk Durhuus^1,2^, Svenja Hansson^1^, Thomas Morville^3^, Anja Birk Kuhlman^3^, Tine Lovsø Dohlmann^3^, Steen Larsen^3,4^, Jørn Wulff Helge^3^, Maria Angleys^1^, Alba Muniesa-Vargas^1^, Jens R. Bundgaard^5^, Ian David Hickson^6^, Flemming Dela^3,7^, Claus Desler^1*^, Lene Juel Rasmussen^1*^

1. University of Copenhagen, Center for Healthy Aging, Department of Cellular and Molecular Medicine, Copenhagen, Denmark

2. The HNPCC register, Clinical Research Center, Copenhagen University Hospital, Hvidovre, Denmark

3 University of Copenhagen, XLAB, Center for Healthy Aging, Department of Biomedical Sciences, Copenhagen, Denmark

4 Clinical Research Centre, Medical University of Bialystok, Bialystok, Poland

5 University of Copenhagen, Department of Clinical Biochemistry, Copenhagen, Denmark

6 University of Copenhagen, Center for Chromosome Stability, Center for Healthy Aging, Department of Cellular and Molecular Medicine, Copenhagen, Denmark

7 Department of Geriatrics, Bispebjerg-Frederiksberg hospital, Copenhagen, Denmark.

*For correspondence: [cdesler@sund.ku.dk](mailto:cdesler@sund.ku.dk) (C.D.) or [lenera@sund.ku.dk](mailto:lenera@sund.ku.dk) (L.J.R.)


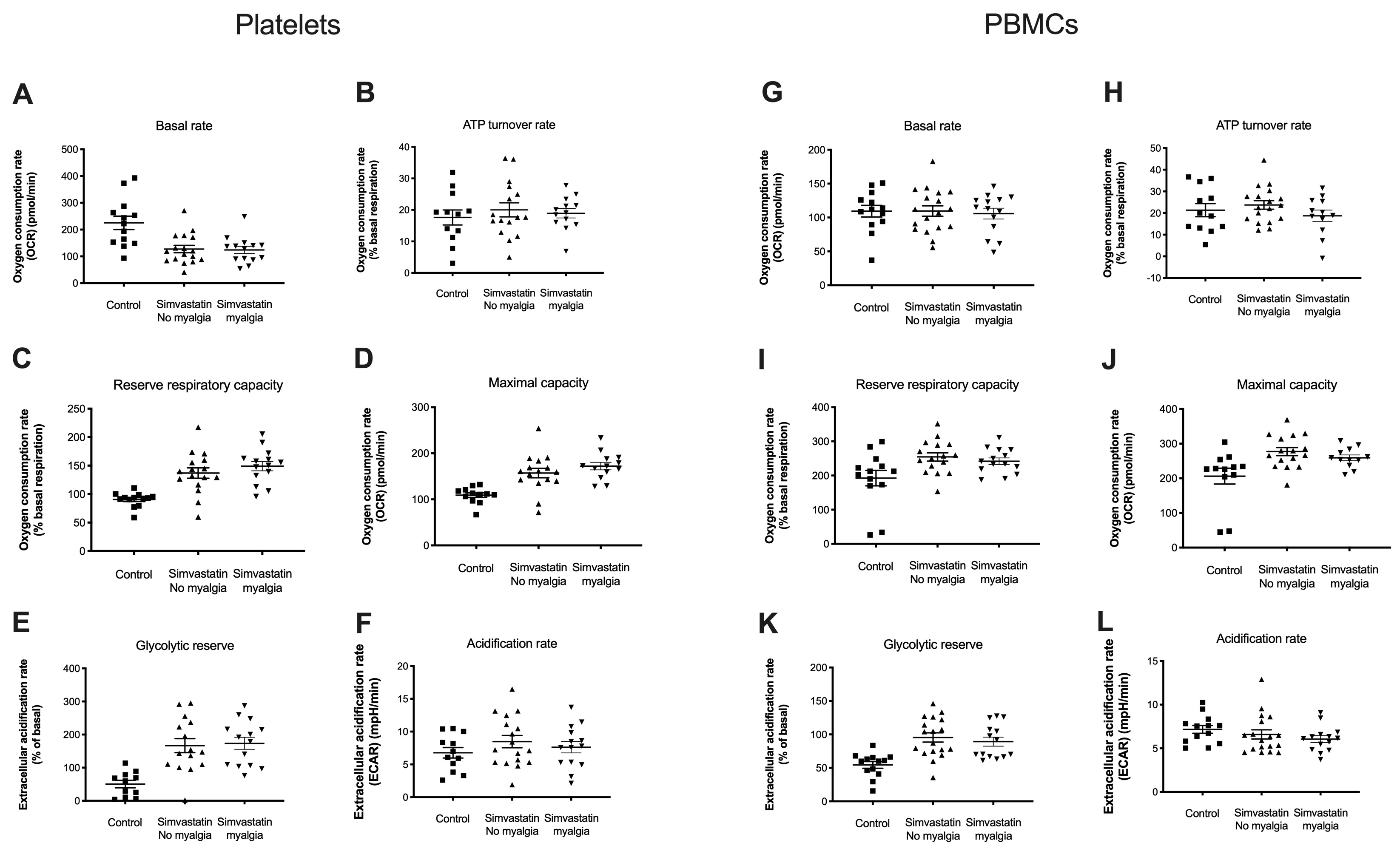


**Figure S1.** Mitochondrial respiratory attributes of control, Simvastatin users with or without myalgia.

**Figure S2.** MtDNA / nDNA ratio between controls and Simvastatin users.


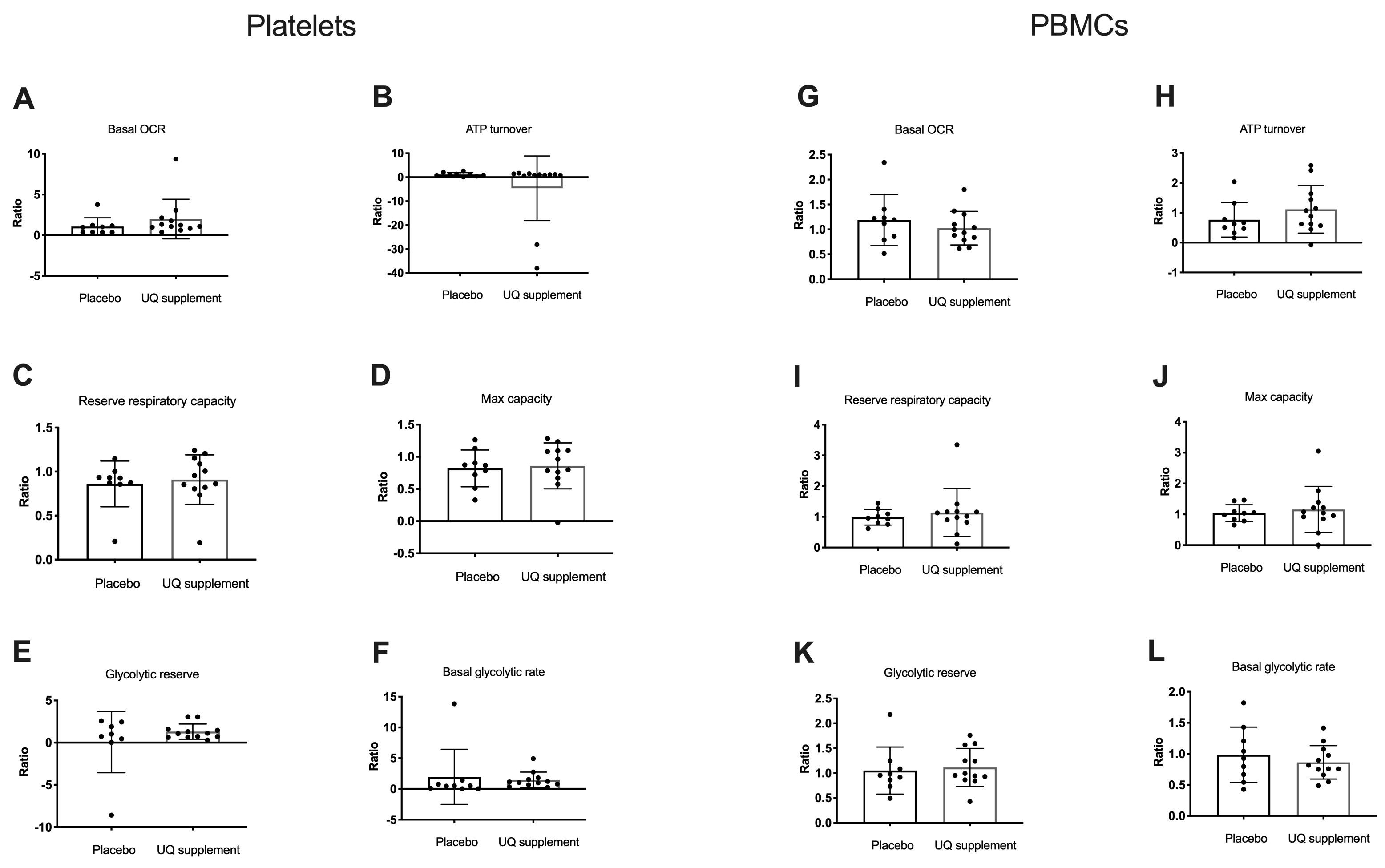


**Figure S3.** Fold difference in parameters of mitochondrial respiration before and after 8-weeks of placebo supplement (left bars) or UQ supplement (right bars)
